# Effects of the F_4_TCNQ-Doped Pentacene Interlayers on Performance Improvement of Top-Contact Pentacene-Based Organic Thin-Film Transistors

**DOI:** 10.3390/ma9010046

**Published:** 2016-01-13

**Authors:** Ching-Lin Fan, Wei-Chun Lin, Hsiang-Sheng Chang, Yu-Zuo Lin, Bohr-Ran Huang

**Affiliations:** 1 Department of Electronic and Computer Engineering, National Taiwan University of Science and Technology, No. 43, Section 4, Keelung Road, Da’an District, Taipei City 106, Taiwan; M10002340@mail.ntust.edu.tw (H.-S.C.); D9802312@mail.ntust.edu.tw (Y.-Z.L.); 2 Graduate Institute of Electro-Optical Engineering, National Taiwan University of Science and Technology, No. 43, Section 4, Keelung Road, Da’an District, Taipei City 106, Taiwan; D10019005@mail.ntust.edu.tw (W.-C.L.); huangbr@mail.ntust.edu.tw (B.-R.H.)

**Keywords:** pentacene, organic thin-film transistors (OTFTs), F_4_TCNQ, Teflon, carrier injection layer

## Abstract

In this paper, the top-contact (TC) pentacene-based organic thin-film transistor (OTFT) with a tetrafluorotetracyanoquinodimethane (F_4_TCNQ)-doped pentacene interlayer between the source/drain electrodes and the pentacene channel layer were fabricated using the co-evaporation method. Compared with a pentacene-based OTFT without an interlayer, OTFTs with an F_4_TCNQ:pentacene ratio of 1:1 showed considerably improved electrical characteristics. In addition, the dependence of the OTFT performance on the thickness of the F_4_TCNQ-doped pentacene interlayer is weaker than that on a Teflon interlayer. Therefore, a molecular doping-type F_4_TCNQ-doped pentacene interlayer is a suitable carrier injection layer that can improve the TC-OTFT performance and facilitate obtaining a stable process window.

## 1. Introduction

Over the past few decades, pentacene-based organic thin-film transistors (OTFTs) have attracted considerable research interest as promising candidates for smart cards, sensors, flexible displays, and radio frequency identification circuitry, because of their unique characteristics, which include their light weight, low-temperature processing, and high mechanical flexibility [1,2,3,4]. Au is usually used as the source/drain (S/D) electrode material in pentacene-based OTFTs since its work function matches the highest occupied molecular orbital level of pentacene; this matching benefits hole injection between the S/D electrodes and the pentacene channel [5]. In a typical top-contact (TC) pentacene-based OTFT structure, the S/D metal electrodes are formed by depositing Au onto the pentacene channel layer. However, when Au is directly deposited onto the pentacene channel layer, it diffuses into the upper layer of pentacene to form a mixture layer of metal and pentacene. The formation of the mixture layer is associated with the formation of hole injection barriers between Au and the pentacene channel layer [6,7,8]. The formation of the hole injection barriers leads to a low carrier injection efficiency and a high contact resistance (*R*_C_), which degrade the performance of TC pentacene-based OTFTs.

In organic light-emitting diodes, the carrier injection efficiency can be enhanced by introducing a carrier injection layer between the organic material and the metal electrode. Introducing a carrier injection layer reduces the barrier height between the organic layer and the metal electrode, increasing the luminance efficiency and current density [9,10]. Thus, in TC pentacene-based OTFTs, enhancement of carrier injection efficiency, which is crucial, can be achieved by introducing a carrier injection layer between the S/D metal electrodes and the pentacene channel layer. The carrier injection layer is believed to play a critical role in improving the electrical characteristics of the TC pentacene-based OTFTs. Carrier injection layers used in TC pentacene-based OTFTs can be divided into two categories: the insulating type of layers, which enables carriers from the S/D metal electrodes to tunnel to the pentacene channel layer easily, and the molecular doping type of layers, which forms a step hole transport configuration and is characterized by high local conductivity, thereby enhancing the injection of holes from the S/D metal electrodes to the pentacene channel layer [7,8,11,12].

The present author have previously reported TC pentacene-based OTFTs fabricated by introducing an ultrathin insulating Teflon carrier injection layer, which enhanced the drain current and field-effect mobility as a result of a decrease in the hole injection barrier and increased tunneling at the Au electrode/pentacene channel interface [7]. However, when the thickness of the Teflon layer exceeds a critical value, the carrier tunneling probability decreases because of an increase in the local resistance at the Au electrode/pentacene channel interface. It was found that the critical thickness of an insulating type of carrier injection layer is less than or equal to 5 nm. This critical thickness value is too low to accurately control the evaporation process, and therefore, it results in an unstable and sensitive process window. In the case of the molecular doping type, the organic materials employed for the buffer layer should be highly conductive for pentacene-based OTFTs, which be attributed to the boost of carrier density. Therefore, the use of the molecular doping materials might be an efficient way to tune carrier injection from metal electrode to organic material, resulting in a conductivity enhancement and thereby improved device performance. Kentaro *et al.* have confirmed that the Fermi energy (*E_F_*) of F_4_TCNQ-doped pentacene layer shifts toward the valence states [13]. Therefore, the molecular doping type of layers can be attributed to the improved conductivity of the p-doped layer with the doping of F_4_TCNQ into the pentacene. Li *et al.* proposed a molecular doping carrier injection layer scheme in which the pentacene carrier injection layer of TC pentacene-based OTFTs is doped with different concentrations of tetrafluorotetracyanoquinodimethane (F_4_TCNQ) [12]. The scheme involves the evaporation of a mixture solution of F_4_TCNQ and pentacene in chloroform with a specific F_4_TCNQ:pentacene ratio. However, the melting point of organic molecules varies with their molecular weight. The melting points of F_4_TCNQ and pentacene are 290 °C and above 300 °C, respectively. The direct evaporation of a mixture solution of F_4_TCNQ and pentacene with a specific F_4_TCNQ:pentacene ratio, rather than the evaporation of these two chemicals individually (*i.e*., simultaneous “co-evaporation”), results in a discrepancy between the practical and designed F_4_TCNQ:pentacene ratios during the evaporation process. The published report indicated that the co-evaporation method also had been used for interlayer deposition between the pentacene channel layer and Au electrodes, such as m-MTDATA-doped V_2_O_5_ [11], MoO*_x_*-doped pentacene [14], and MoO_3_ and m-MTDATA-doped pentacene [15]. However, the co-evaporation scheme of the F_4_TCNQ and pentacene for forming an F_4_TCNQ-doped pentacene interlayer had been not investigated.

In this study, for the first time, the co-evaporation scheme is used for evaporating F_4_TCNQ and pentacene simultaneously to form an F_4_TCNQ-doped pentacene interlayer between the Au S/D electrodes and the pentacene channel layer of TC pentacene-based OTFTs and, thus, improve the OTFT performance. The field-effect mobility was enhanced by a factor of 1.6 and the on/off current ratio was enhanced by a factor of 1.5 compared with a control device. In addition, the influence of the F_4_TCNQ-doped pentacene interlayer thickness on the electrical characteristics of the OTFTs was investigated. The dependence of the device performance on the F_4_TCNQ-doped pentacene interlayer (molecular doping type) thickness was not stronger than that for a Teflon interlayer (insulating type). Therefore, the F_4_TCNQ-doped pentacene layer is a suitable carrier injection layer between the Au electrodes and the pentacene channel layer because it can not only improve the device performance but also facilitate obtaining a larger and stable process window.

## 2. Device Fabrication

**Figure 1:**
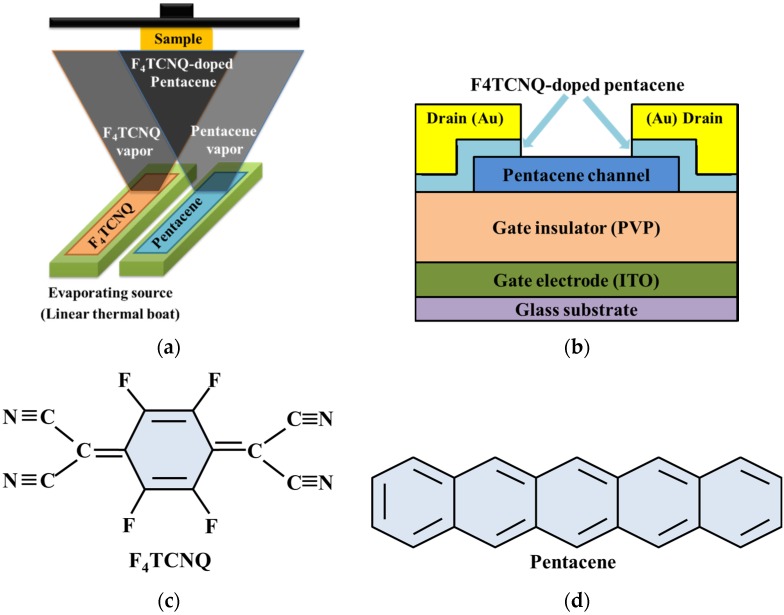
(**a**) a schematic diagram of the co-evaporation process; (**b**) a schematic structure of the pentacene-based organic thin-film transistors (OTFTs) with F_4_TCNQ-doped pentacene injection layer, and the molecular structures of (**c**) F_4_TCNQ and (**d**) pentacene, respectively.

Pentacene-based OTFTs with a bottom-gate and top-contact structure were fabricated. A glass substrate with an indium-tin-oxide (ITO) layer was used as the substrate and as the bottom-gate electrode. A 350-nm-thick cross-linked poly (4-vinylphenol) (PVP) coating was provided on the ITO layer for use as the gate dielectric, and it was cured in a vacuum oven at 200 °C for 1.5 h. A 50-nm-thick pentacene channel layer was then deposited on the PVP layer through a shadow mask by using a thermal evaporator (base pressure: 2.0 × 10^−6^ Torr) at a deposition rate of 0.2 Å/s and with the substrate temperature maintained at room temperature. Before the deposition of the S/D metal electrodes, a 1.5-nm-thick F_4_TCNQ-doped pentacene carrier injection interlayer was deposited on the pentacene channel layer through a metal mask by simultaneously co-evaporating F_4_TCNQ and pentacene from a thermal evaporator. In the co-evaporation process, the source materials of pentacene and F_4_TCNQ were independently controlled, and were heated by linear thermal boat when the materials are deposited, as shown in Figure 1a. The deposition rate of F_4_TCNQ was fixed at 0.1 Å/s, while that of pentacene was set at 0.1, 0.3 and 1.0 Å/s; the designed F_4_TCNQ:pentacene ratios corresponding to the pentacene deposition rates were 1:1, 1:3 and 1:10 respectively. Finally, Au (50 nm) S/D electrodes were formed through a shadow mask by using a thermal evaporator. Therefore, the F_4_TCNQ-doped pentacene can be uniformly deposited to form the interlayer between pentacene channel layer and Au metal electrodes. The width and length of the electrode channels were 500 and 100 μm, respectively. Figure 1b shows a schematic structure of the pentacene-based OTFTs with F_4_TCNQ-doped pentacene interlayer, and Figure 1c,d show the molecular structures of F_4_TCNQ and pentacene, respectively. For convenience, the devices with F_4_TCNQ:pentacene ratios of 1:1, 1:3 and 1:10 are hereafter designated as Devices 1, 2 and 3, respectively. A device without an F_4_TCNQ-doped pentacene interlayer was considered the control sample. All measurements were performed under dark conditions and at an ambient temperature by using a semiconductor parameter analyzer (HP 4145B, HP Inc., Palo Alto, CA, USA).

## 3. Results and Discussion

**Figure 2:**
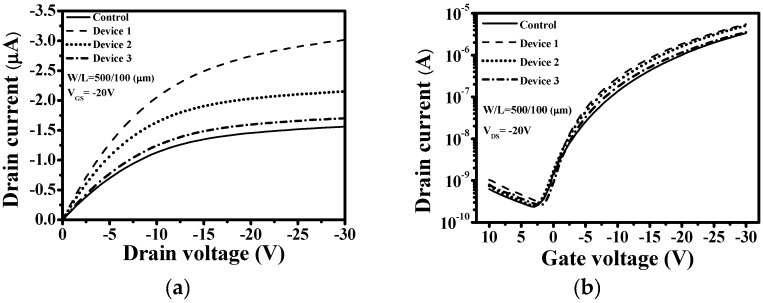
Drain current characteristics of top-contact (TC) pentacene-based OTFTs with different F_4_TCNQ:pentacene ratios (Device 1 = 1:1, Device 2 = 1:3, and Device 3 = 1:10) (**a**) Output curves (*I*_DS_-*V*_DS_). (**b**) Transfer curves (*I*_DS_-*V*_GS_).

Figure 2a shows the output characteristics (*I*_DS_-*V*_DS_) of the devices with different F_4_TCNQ:pentacene ratios for a *V*_GS_ = −20 V. The saturation current of Device 1 was −3.02 × 10^−6^ A for *V*_DS_ and *V*_GS_ values of −30 and −20 V, respectively; this saturation current was much higher than those of Devices 2 and 3 and the control sample. The *I*_DS_ value of Device 1 was approximately 193% higher than that of the control sample. Figure 2b shows the transfer characteristics (*I*_DS_-*V*_GS_) of the devices with different F_4_TCNQ:pentacene ratios. The μ_FE_ value of Device 1 was enhanced by a factor of 1.6 and the on/off current ratio was enhanced by a factor of 1.5 compared with the control sample. This appreciable improvement in the electrical performance of Device 1 could be attributed to the F_4_TCNQ:pentacene ratio being appropriate and to the charge density at interface between the S/D electrodes and the pentacene channel being high, improving charge injection [12,16]. However, the μ_FE_ value of the devices decreased with a decrease in the F_4_TCNQ concentration of the F_4_TCNQ-doped pentacene interlayer.

**Figure 3:**
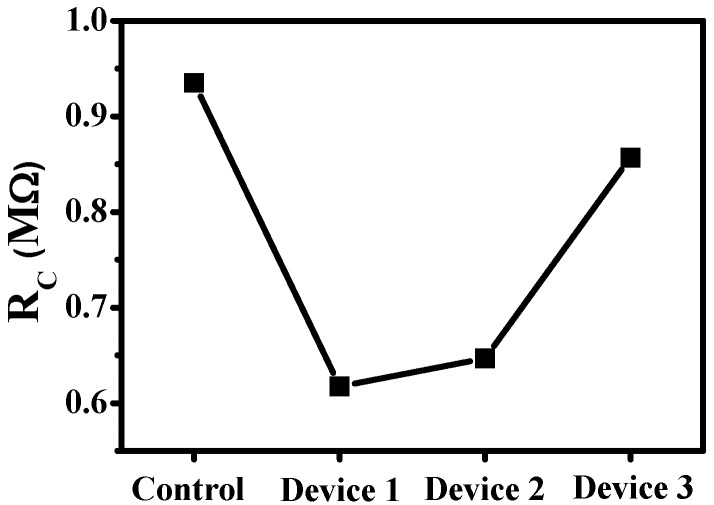
The *R*_C_ value as a function of F_4_TCNQ:pentacene ratio of TC pentacene-based OTFTs for *V*_DS_ and *V*_GS_ values of −2 and −20 V, respectively.

To quantitate the effect of the F_4_TCNQ:pentacene ratio of the F_4_TCNQ-doped pentacene interlayer on the electrical contact between the pentacene channel layer and the S/D metal electrodes, the channel length dependence of the total resistance (*R*_Total_) was determined according to the linear regions of the output characteristics of the fabricated devices. The channel lengths were 100, 150, 200 and 250 μm. The *R*_C_ value was obtained from the transmission line model (TLM) by extrapolating the *R*_Total_ curve to obtain the *y*-intercept for zero channel length [17]. The *R*_C_ values of the four devices, determined from the TLM, are shown in Figure 3. The *R*_C_ value of Device 1 at a gate voltage of −20 V was 0.617 × 10^6^ Ω, which is approximately 1.5 times lower than that of the control sample (0.935 × 10^6^ Ω). As shown in Figure 3, *R*_C_ first decreases and then increases with a decrease in the F_4_TCNQ concentration of the F_4_TCNQ-doped pentacene interlayer. Apparently, the F_4_TCNQ concentration affects hole transport across the interface between the S/D metal electrodes and the pentacene channel layer. In the presence of an F_4_TCNQ-doped pentacene interlayer, the hole carrier could easily move through the F_4_TCNQ-doped pentacene layer into the pentacene channel because of the improvement in the local conductivity and the formation of a multiple step barrier at the interlayer [12,16]. F_4_TCNQ is an acceptor dopant, and therefore, in the interlayer, it leads to which hole doping involving the electron transfer of energy level between pentacene and F_4_TCNQ, resulting in enhancing conductivity. Furthermore, the F_4_TCNQ-doped pentacene interlayer changes the Fermi energy (*E*_F_) position of the intrinsic pentacene, which leads to the formation of subdivided barriers instead of a single barrier in the interlayer, and enhances the injection of hole carriers from the Au electrodes to the pentacene channel. Consequently, the F_4_TCNQ dopant amount can be easily fine-tuned to achieve the optimal device performance, which corresponds to a reduction in *R*_C_, as shown in Figure 3.

**Figure 4:**
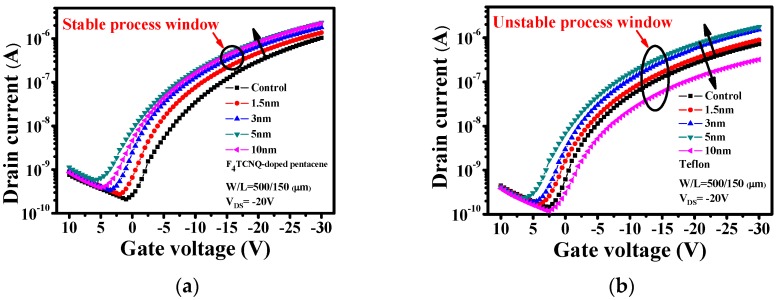

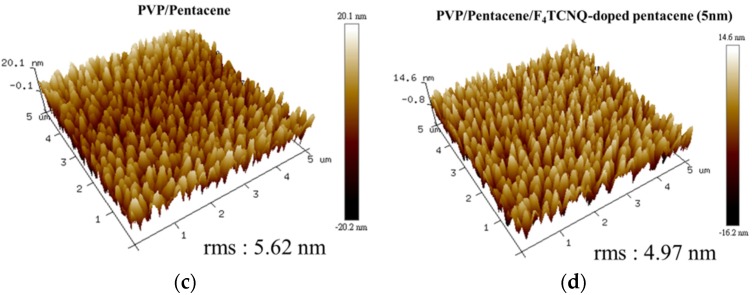
Transfer characteristics of pentacene-based OTFTs with (**a**) F_4_TCNQ-doped pentacene and (**b**) Teflon interlayers of various thicknesses, and AFM images of pentacene films (**c**) without and (**d**) with 5 nm F_4_TCNQ-doped pentacene interlayer.

The dependence of the device performance on the thickness of the molecular-doping-type F_4_TCNQ-doped pentacene layer was investigated. In a previous report of the present authors, the performance of pentacene-based OTFTs was found to be strongly dependent on the thickness of the insulating-type Teflon interlayer [7]. To investigate the effect of the thickness of F_4_TCNQ-doped pentacene and Teflon interlayers on the device performance, the F_4_TCNQ:pentacene ratio of the F_4_TCNQ-doped pentacene interlayer was fixed at 1:1, and the thicknesses of both carrier injection interlayers were set at 0, 1.5, 3, 5 and 10 nm. Figure 4a,b shows the thickness-dependent transfer property of the device with Teflon and F_4_TCNQ-doped pentacene interlayers of various thicknesses. The device with Teflon interlayer showed remarkable thickness dependence, while the one with F_4_TCNQ-doped pentacene interlayer showed a slight dependence. In addition, Figure 4c,d shows the AFM micrographs of the pentacene layers without and with a 5 nm F_4_TCNQ-doped pentacene interlayer deposited on the PVP dielectric surface. The root mean square (rms) roughness for both devices without and with 5 nm F_4_TCNQ-doped pentacene interlayer is 5.62 and 4.97 nm, respectively. It had been clearly seen that the surface become more smooth for devices with 5 nm F_4_TCNQ-doped pentacene interlayer, which is necessary for providing a better contact with metal electrodes. This is the mechanism for lowering the barrier height and increasing the charge injection, and then improving device performance. The field-effect mobility (μ_FE_) was calculated for a *V*_GS_ value of −30 V in the saturation region, and the threshold voltage (*V*_TH_) was determined according to the saturation region of the plot of **|***I*_DS_**|**^1/2^ *versus* *V*_GS_ [18]. The sub-threshold swing (*SS*) was calculated using the equation *SS* = *dV*_GS_/*d*(log_10_**|***I*_DS_**|**). The maximum and minimum values of *I*_DS_ at a *V*_DS_ of −20 V were designated as *I*_ON_ (on current) and *I*_OFF_ (off current), respectively [19]. The thickness-dependent properties of pentacene-based OTFTs with Teflon and F_4_TCNQ-doped pentacene interlayers of various thicknesses were summarized in Table 1.

**Table 1:** The electrical characteristics for the devices with F_4_TCNQ-doped pentacene and Teflon interlayers of various thicknesses.

Electrical Parameters	Molecular Doping Type: F_4_TCNQ-Doped Pentacene					Insulating Type: Teflon				
	Control	1.5 nm	3 nm	5 nm	10 nm	Control	1.5 nm	3 nm	5 nm	10 nm
*V*_TH_ (V)	−7.7	−5.7	−4.4	−4.1	−4.2	−6	−5.9	−5.5	−3	−5.3
*SS* (V/decade)	2.2	2.7	2.5	2.6	2.9	1.77	1.98	1.73	1.93	2.5
μ_FE_ (cm^2^·V^−1^·s^−1^)	0.12	0.14	0.17	0.21	0.2	0.08	0.09	0.12	0.15	0.02
*I*_ON_/*I*_OFF_ (10^3^)	4.95	5	5.34	4.3	5.76	10.4	9.86	20.2	15.6	39.7

**Figure 5:**
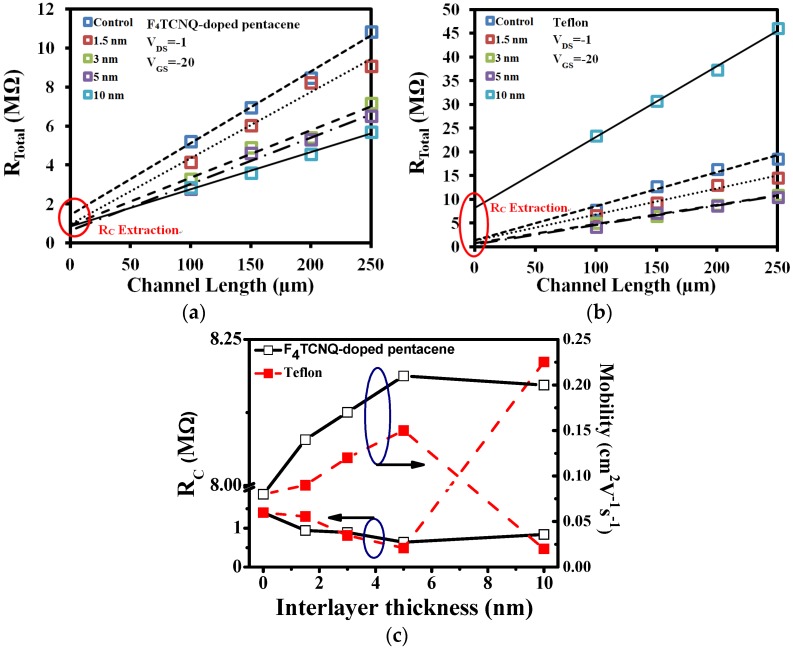
The *R*_Total_ and *R*_C_ of pentacene-based OTFTs with the different thickness of the (**a**) F_4_TCNQ-doped pentacene and (**b**) Teflon interlayer. (**c**) The variation of the mobility and *R*c values with the various carrier-injection interlayer thickness for the devices with F_4_TCNQ-doped pentacene or Teflon interlayers.

The *R*_Total_ of the device with Teflon and F_4_TCNQ-doped pentacene interlayers of various thicknesses is shown in Figure 5a,b, respective. The variation of the *R*_C_ and μ_FE_ values with the carrier-injection interlayer thickness for the devices with Teflon and F_4_TCNQ-doped pentacene interlayers is shown in Figure 5c. The *R*_C_ value of the device with a molecular-doping-type F_4_TCNQ-doped pentacene interlayer depended only slightly on the F_4_TCNQ-doped pentacene interlayer thickness. The performances of both types of devices, according to μ_FE_, increased with the thickness. However, when the interlayer thickness exceeded 5 nm, the μ_FE_ value of the device with an F_4_TCNQ-doped pentacene interlayer decreased slightly; by contrast, the μ_FE_ value of the device with a Teflon interlayer decreased appreciably. It is presumed that the degradation of μ_FE_ can be directly attributed to the increase in *R*_C_. Unlike the *R*_C_ value of the device with an insulating-type Teflon interlayer, the *R*_C_ value of the device with a molecular-doping-type F_4_TCNQ-doped pentacene interlayer depended only slightly on the F_4_TCNQ-doped pentacene interlayer thickness. Teflon is an insulating material with an extremely high resistivity (10^18^ Ω·cm). Therefore, when the Teflon interlayer thickness exceeds a critical value, the insulating properties of the interlayer become dominant and reduce the carrier tunneling probability. Consequently, the device performance is degraded appreciably. It is to say that the molecular doping-type F_4_TCNQ-doped pentacene interlayer is a suitable carrier injection layer that can not only improve the TC-OTFT performance but also facilitate obtaining a stable process window. It is the first time to compare the process windows for the interlayers of the insulating type and molecular-doping type. It is believed that the results will be interesting and important from the viewpoint of manufacturing as a result of the stable process window.

**Figure 6:**
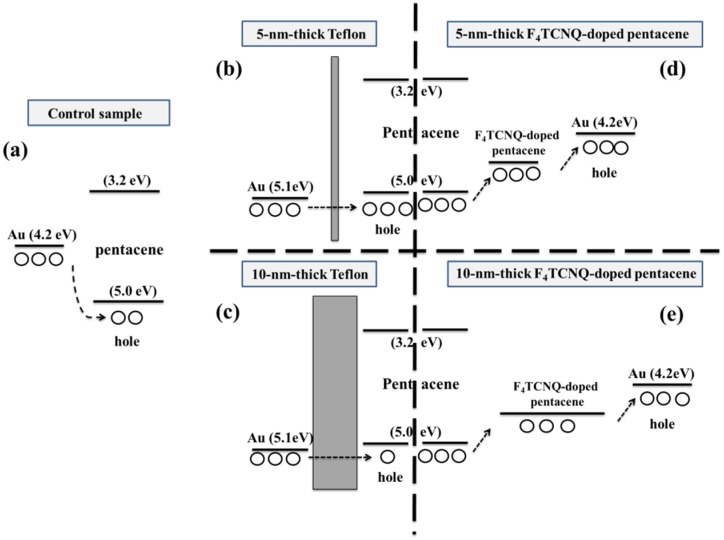
Band diagram of top-contact (TC) pentacene-based OTFTs with various interlayer thicknesses (**a**) control sample, (**b**) 5-, and (**c**) 10-nm-thick Teflon and (**d**) 5-, and (**e**) 10-nm-thick F_4_TCNQ-doped pentacene.

In Figure 6, the dependence of the carrier injection efficiency on the thickness of the Teflon and F_4_TCNQ-doped pentacene is clearly explained. In the control sample, hot Au atoms are directly deposited onto the pentacene channel layer, and diffuse into the upper layer of pentacene. A high energy barrier formed because of the presence of an interface dipole, there is a hole injection barrier of 0.8–1 eV between the Au electrodes and the pentacene channel layer, resulting in a relatively large hole injection barrier, as shown in Figure 6a [6,7]. In addition, a high energy barrier associated with the formation of a metallic surface at the Au electrode/pentacene channel interface obstructed hole carrier transport. In the 5-nm-thick Teflon interlayer, the carriers could easily tunnel through to the pentacene channel layer because of suppressed metal penetration and a reduced hole injection barrier at the Au electrode/pentacene channel interface [7]. However, for a Teflon layer thickness greater than 5 nm, the probability of carriers tunneling through clearly decreased because of the increased *R*_C_ at the Au electrode/pentacene channel interface. In the case of the F_4_TCNQ-doped pentacene interlayer, regardless of its thickness (5- or 10-nm thick), the hole carriers could easily move through the interlayer to the Au electrodes because of the formation of a subdivided barrier and high local conductivity at the Au electrode/pentacene channel interface, which effectively reduced the hole injection barrier. Moreover, in the device containing a 5-nm-thick F_4_TCNQ-doped pentacene interlayer with an F_4_TCNQ:pentacene ratio of 1:1, the carrier injection efficiency is high and *R*_C_ is low, resulting in high device performance. Even when the thickness of the F_4_TCNQ-doped pentacene interlayer was 10 nm, the effect of the thickness on the OTFT electrical performance was not apparent, implying that the thickness dependence of the electrical performance of OTFTs with a molecular-doping-type F_4_TCNQ-doped pentacene interlayer is weaker than that of the performance of OTFTs with an insulating-type Teflon interlayer. Therefore, it is believed that an F_4_TCNQ-doped pentacene interlayer is a good candidate as a carrier injection layer for TC pentacene-based OTFTs since it provides a high carrier injection efficiency and a stable process window.

## 4. Conclusions

In summation, An F_4_TCNQ-doped pentacene interlayer was fabricated as a carrier injection layer by using the co-evaporation method, and the effect of the doping concentration and thickness of the interlayer on the performance of pentacene-based OTFTs was investigated. The results show that the device with an F_4_TCNQ:pentacene ratio of 1:1 showed considerably improved electrical characteristics compared with the device without an interlayer, because of a decrease in the contact resistance between the metal electrodes and the pentacene channel. Furthermore, observations suggested that the dependence of the electrical performance of the OTFTs on the molecular-doping-type F_4_TCNQ-doped pentacene interlayer thickness was weaker than that of the performance of an insulating-type Teflon interlayer. Therefore, it is believed that the F_4_TCNQ-doped pentacene interlayer (molecular doping type) is a suitable candidate for the carrier injection layer of TC pentacene-based OTFTs since it facilitates obtaining a stable process window and improves the OTFT performance.
